# The experience of buprenorphine implant in patients with opioid use disorder: a series of narrative interviews

**DOI:** 10.3389/fpsyt.2023.1205285

**Published:** 2023-08-31

**Authors:** Pietro Scurti, Marco Nunzi, Claudio Leonardi, Claudio Pierlorenzi, Roberta Marenzi, Vincenzo Lamartora

**Affiliations:** ^1^ASL Napoli 2 Nord, Napoli, Italy; ^2^UOS Patologie da Dipendenza d9 ASL Roma 2, Roma, Italy; ^3^ASST Papa Giovanni XXIII, Ospedale di Bergamo, Bergamo, Italy

**Keywords:** buprenorphine implant, narrative medicine, opioid agonist therapy, opioid use disorder, quality of life

## Abstract

**Background:**

This study used narrative medicine (NM) to assess the point of view of patients with opioid use disorder (OUD) and the impact that addiction and a new treatment approach via buprenorphine implant had on their daily lives as compared with previous oral Opioid Agonist Therapy (OAT).

**Methods:**

Five patients with OUD undergoing treatment with a buprenorphine subcutaneous implant participated voluntarily and provided their anonymity by self-describing, in response to questions prompted by the clinician, their experience with this innovative therapy. The narratives were analyzed according to standard NM methodology. Citations of patients' positive or negative experiences with traditional OAT and buprenorphine implant were classified according to five categories—patient's determination toward complete opioid abstinence, emotional impact, impact on life, smoothness of therapy, and therapy dependency—and quantified to obtain a picture of the overall therapy experience.

**Results:**

The analysis revealed the extent of the burden not only of addiction but also of the traditional OAT on patients' life, including relationships with family, job management, and free time. Conversely, the therapy with buprenorphine implant revealed a significant improvement in the quality of life of the patients, who also largely reported a positive emotional outcome during this therapy, as well as a solid determination to achieve complete recovery.

**Conclusions:**

This study illustrates the complex problems of living with OUD and provides insights into the added value of an innovative buprenorphine implant therapy that, due to its administration route and prolonged duration, allows patients to take an additional step toward total opioid abstinence and complete recovery of daily life.

## 1. Introduction

In 2020, ~284 million people aged 15–64 years used drugs worldwide, while nearly 39 million people suffered from drug use disorders (1). Globally, it is estimated that more than 61 million individuals are opioid abusers, i.e., 1.2% of the world's population, thus opioids continue to be the largest contributor to disabilities and mortality attributed to drug use. Opioid use disorder (OUD) refers to the long-term compulsive self-administration of opioids for non-medical purposes (1, 2). OUD is a chronic disorder at high risk of relapse that, although initially driven by the activation of reward brain circuits, progressively involves more and more “anti-reward” related circuits, which in turn drive negative emotional states and relapse. However, recovery is possible with appropriate treatment, albeit with a persistent propensity to relapse (3–5).

Opioid agonist therapy (OAT) occupies and activates opioid receptors, relieving withdrawal symptoms and reducing cravings (4, 6, 7). The two most common opioid agonists are methadone and buprenorphine, which are often formulated for oral intake and are recommended as first-line interventions in the NICE guidelines (8). Unfortunately, opioid-based oral medications are often diverted (i.e., traded by illicit/unconventional means) or misused (i.e., employed beyond the therapeutic indications), making OAT management particularly critical even beyond the individual patient (4, 7, 9). Moreover, cases of accidental ingestion—which may also involve minors—are not infrequent, often resulting in severe overdose reactions. The access to oral OAT is necessarily mediated by the Addiction Service (Ser.D. in Italian, from “*Servizio Dipendenze*”), where one must regularly go in person to receive treatment, therefore limiting the planning of daily activities (including work), compromising the establishment of a “normal” routine, hindering off-site activities (such as vacations), and ultimately causing lack of adherence to therapy. Finally, social stigma is a highly relevant limiting factor in new patients' access to Ser.D.

In light of the above, innovative medium- or long-acting formulations—such as implants or depots—can counteract stigma by dramatically decreasing the need to visit Ser.D.; moreover, they can increase adherence through certainty of therapy uptake and by reducing their intrusiveness in the patient's life (4, 10). Long-lasting OAT formulations allow as well to reach more stable plasma levels of the drug and may favor the rapid achievement of an optimal quality of life (4, 10). The FDA is promoting the application of abuse-deterrent OAT, to counteract misuse and diversion (11). Among recent innovative long-acting formulations, the buprenorphine implant, intended to remain in place on the inner side of the arm releasing the drug continuously for 6 months, is revealing its efficacy (3). FDA approved buprenorphine implant for OUD treatment in 2016, whereas the date of issue of marketing authorization valid throughout the European Union was on June 2019 (12, 13). In Italy, buprenorphine implant is already on the market, and the first clinical experiences of patients at the European level are being collected.

To explore the impact of buprenorphine implants on patients' quality of life, we addressed them directly via narrative medicine (NM), an approach that allows patients to describe their experience through a semi-guided interview process, in written or oral form (14). According to one of the definitions available in the literature, NM “is rewriting medical and scientific terminology so that it is more consistent with the patient's experience and thinking” (15). In the context of OAT, NM allows one to freely gather insights about the benefits/disadvantages of therapy (e.g., freedom, work, stigma, social life, etc.), management of daily routine, quality of healthcare service, differences with previous therapy, and other factors that may impact and determine the long-term success of therapy.

In this study, we report the interviews of five patients who had been using buprenorphine implants for at least 3 months. The methodology of NM was implemented via a semi-guided interview by prompting patients' narratives through planned questions, as well as via qualitative content analysis, as based on previous literature (16–19). This study aimed to collect and compare patients' experiences before (during the time they were treated with daily oral OAT) and after the subcutaneous implant, through their narratives.

## 2. Materials and methods

### 2.1. Participants

Five participants, all male, aged >18 years, former opioid abusers with previous experience of oral OAT, were recruited directly from their referring clinicians at the Ser.D.—doctors and psychologists—and were invited to share their ongoing experience with buprenorphine implant. Patients were recruited in different regions of Italy (Campania, Lazio, Lombardia), and the interviews took place between October and November 2022. After the surgical insertion of the buprenorphine implant, patients were monitored through regular visits. Overall, most patients were visited weekly (in the first month), then fortnightly (in the second month), and then monthly. Regular toxicological tests were performed to evaluate the possibility of intake of illegal opioids; all tests were found to be negative for opioids.

### 2.2. Ethical considerations

Participants agreed to be interviewed by their Ser.D. contact persons, at their respective locations, anonymously, after reading and signing the informed consent according to the ethical guidelines of the Declaration of Helsinki (20) and to the most recent European General Data Protection Regulation (21). Two participants consented to a video interview, two consented to an audio recording, and one responded in written form. Patients thus shared their stories anonymously, and references to elements that could lead to their identification, either in the transcripts of the oral interviews or in the interviews in written form, were removed by the clinicians.

### 2.3. Buprenorphine implant

A buprenorphine implant is a medicinal product consisting of four rods (26.5 mm × 2.4 mm) each containing buprenorphine hydrochloride equivalent to 74.2 mg buprenorphine. The buprenorphine implant is indicated for the treatment of OUD in clinically stable, opioid-tolerant adult patients who require no more than 8 mg/day of sublingual buprenorphine as OAT. The implant is inserted in the inner side of the arm and is meant to remain in place for 6 months, releasing buprenorphine continuously. Following the insertion procedure, an initial peak of buprenorphine occurs. The median maximum plasmatic concentration is reached after ~12 h from the insertion procedure, then buprenorphine plasmatic concentration gradually decreases up to a steady state of about 0.5–1 ng/ml, approximately after 4 weeks, and is maintained up to week 24. Buprenorphine concentrations delivered by the subcutaneous implant are comparable with the minimum plasmatic concentration of sublingual buprenorphine at doses of 8 mg per day (22–24).

### 2.4. Semi-structured interview

The narrative interview, in semi-guided form, included 20 questions. Patients explored, at first, childhood and preadolescence as well as their first encounter with drugs. These first questions aimed at understanding the patient's background, besides putting the patients at ease and getting them acclimated to the interview setting and structure. Next, patients described their encounters with Ser.D. and their experience with traditional OAT. Afterward, questions explored patients' experience with buprenorphine implants and the related emotional experience, constraints, and advantages. Finally, the last questions of the interview delved into the patient's interpretations, perceptions, and concluding observations of the overall journey. For the full text of the semi-guided interview, see Appendix I.

### 2.5. Analysis of the narrative

A narrative analysis was carried out on each interview. Throughout each transcript, we identified recurrent common topics emerging from their answers that related to the main themes explored (*qualitative analysis*). Furthermore, relative to the description of traditional OAT and buprenorphine implants, we noted and quantified in frequency quotations concerning the strengths and disadvantages of both therapies (*semi-quantitative analysis*).

#### 2.5.1. Qualitative analysis

The five transcripts were scrutinized and the main themes were highlighted. Following a qualitative evaluation, common recurring topics were identified and reported in the results section. Where relevant, the number of patients who explicitly referred to one of the identified topics was reported in the corresponding supplemental tables where relevant quotes are exemplified.

#### 2.5.2. Semi-quantitative analysis

A semi-quantitative approach was adopted relative to the strengths and disadvantages of traditional OAT and buprenorphine implant. Specifically, from the transcript of each interview, we analyzed statements that denoted a reference to the impact of these two types of treatment on patients' quality of life. All quotes about patients' experience with one or the other treatment, separately, were included in the analysis and then assigned to one or more of the following categories: 1) determination toward complete opioid abstinence, 2) emotional impact, 3) impact on life (time, money, work, and social life), 4) smoothness of therapy, and 5) therapy dependency. Next, the positive or negative valence was noted for each statement. Two independent coders selected the quotes to be analyzed, assigned each quote to the appropriate category(ies), and noted the positive or negative valence for each quote. The two independent coders agreed on more than 90% of the category assignment of the scrutinized quotes.

##### 2.5.2.1. Determination toward complete opioid abstinence

To this category were assigned statements that denoted the patient's intention to adhere to the OUD recovery course, such as “I want to quit” (positive value) or “I have not always been assiduous” (negative value).

##### 2.5.2.2. Emotional impact

To this category were assigned statements that referred to clearly identifiable emotions (e.g., anger, happiness, depression, etc.), such as “I was just fine” (positive value) or “it made me angry” (negative value).

##### 2.5.2.3. Impact on life (time, money, work, and social life)

To this category were assigned statements that referred to time, money, work, and social life, such as “I had a great vacation” (positive value) or “it compromises work relationship and daily routine” (negative value).

##### 2.5.2.4. Smoothness of therapy

To this category were assigned statements that denoted possible intervening problems (including “craving”) or smoothness of events, such as “it never bothered me to go to the Ser.D.” (positive value) or “I found the Ser.D. an unpleasant context” (negative value).

##### 2.5.2.5. Therapy dependency

To this category were assigned statements that denoted a sense of freedom or dependence toward therapy, such as “I did not have to go to Ser.D.” (positive value) or “you depend on others (i.e., Ser.D. staff; Editor's note)” (negative value).

## 3. Results

### 3.1. Life background—qualitative analysis

#### 3.1.1. Childhood

The first area covered by the interview explored patients' childhood (see Supplemental Table 1). The five participants were found to be heterogeneous in terms of cultural, social, and family backgrounds; however, they all described an overall normal childhood, with a few difficulties arising from either parental expectations, family conflicts, or racial difficulties. Most patients reported positive experiences during the **schooling time**, except one. Two patients reported doing very well in school and one of them was perceived highly by his family. Positive experiences also often emerged from the **family** theme: families are indeed mostly described as normal. One of the patients was adopted and did not report family issues; another was born into a family with two half-siblings, with whom he got along well. Two patients reported growing up in wealthy families, and one described himself as introverted. Three patients made direct reference to a sense of **happiness** during childhood. Three patients directly expressed a feeling of normality, whereas only one of the patients, of African descent, reported experiencing **discrimination**, but he also described solid friendships. Many patients referred to a past of good sociability, apart from one who described difficulties only in middle school. A summary of patient narratives is available in Appendix II.

#### 3.1.2. The encounter with opioids

Regarding the time, situation, and life context in which participants started abusing opioids, the responses were rather heterogeneous, although some shared similarities emerged (see Supplemental Table 2). Concerning the age of opioid use initiation, this ranged from early years of university to about 30 years. All patients said they had used cannabis (daily or occasionally) during their life, and some of them also experienced other drugs. At least three of the interviewees reported that they were going through some mental distress when they started abusing drugs (e.g., loneliness and separation from wife). At least two patients explicitly mentioned that they decided to try heroin out of naivety or lack of knowledge, two did it because of the social context, and two mentioned that they tried it “for fun.” Finally, one mentioned that he did it “to feel rebellious, non-conformist.”

Although all patients mentioned a rewarding sense of euphoria as an immediate effect of opioid use, all of them suffered serious consequences that led them to Ser.D. (see Supplemental Table 3). At least four patients cited explicitly a feeling of hopelessness, despair, or suffering, and three described a strong sense of dependence. At least two patients mentioned work or school problems, and one lost his job. On the relational side, two patients reported a sense of disconnection and estrangement. Moreover, at least two patients mentioned financial problems, among the reasons that led them to approach Ser.D. A summary of patient narratives is available in Appendix II.

### 3.2. Experience with traditional OAT—semi-quantitative analysis

Following the encounter with Ser.D., all patients were prescribed OAT. Oral OAT duration was variable among participants, ranging from 10 months to 23 years. Some patients cited both methadone and buprenorphine as OATs throughout their treatment history, with varying dosages over time. The highest buprenorphine dose cited corresponded to 28 mg, while the lowest dose corresponded to 0.5 mg. Most patients also mentioned individual or community psychotherapy concomitantly to OAT.

**Figure 1:**
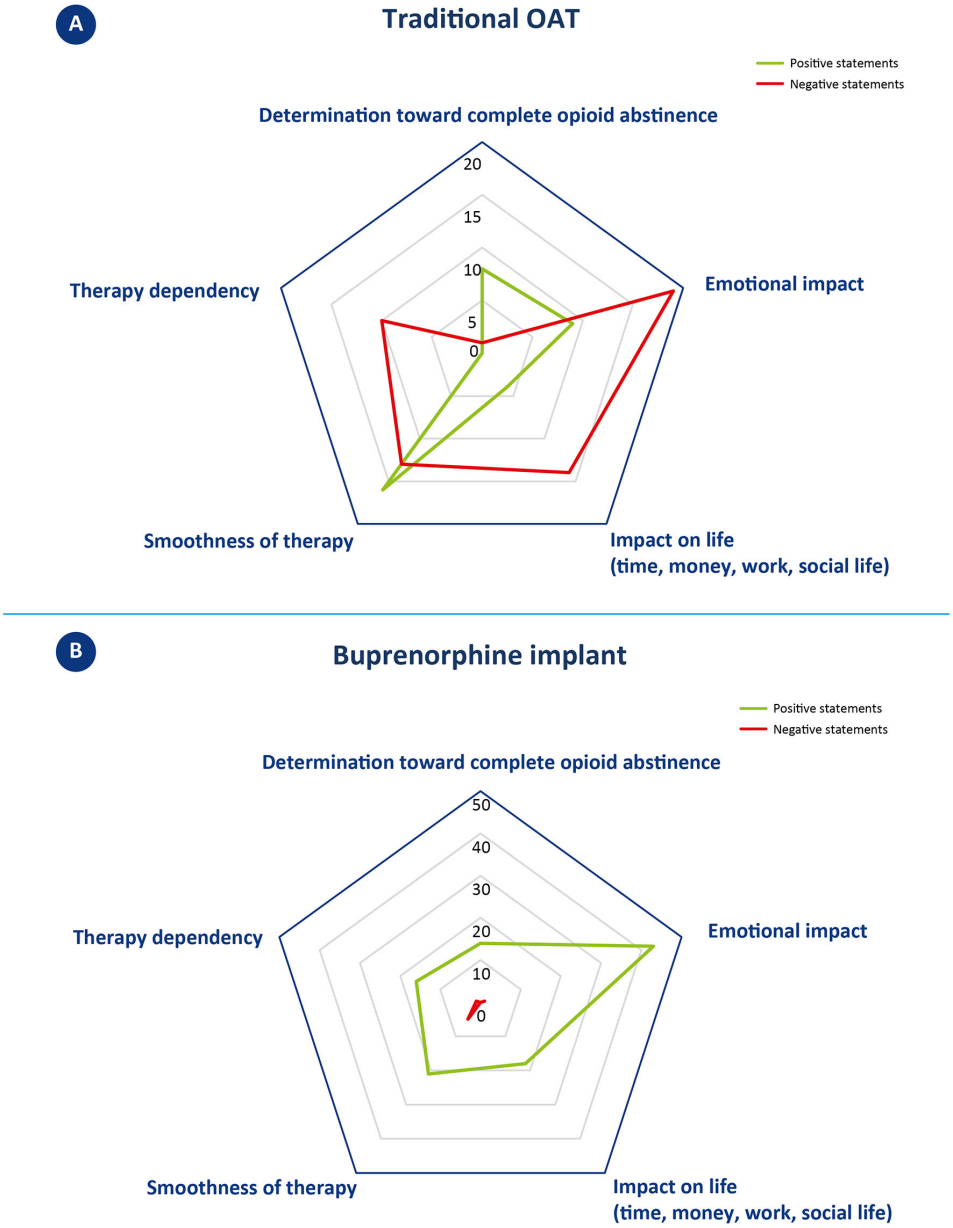
Narrative analysis of patients' reported experience with traditional OAT **(A)** and buprenorphine implant **(B). (A)** Statements concerning oral OAT were classified in one or more of the five identified topics located in the vertices of the pentagon. The number of positive (green line) and negative (red line) statements per topic are plotted along the direction of the corresponding vertex and connected by a 5-pointed closed line. The greater the distance from the center, the larger the number of iterations. **(B)** Statements concerning buprenorphine implant were classified in one or more of the five identified topics located in the vertices of the pentagon; examples of positive or negative statements are reported in the corresponding oval. The number of positive (green line) and negative (red line) statements per topic are plotted along the direction of the corresponding vertex and connected by a 5-pointed closed line. The greater the distance from the center, the higher the number of iterations. Please note that here the scale of the pentagon is different from the one reported in A.

**Figure 2:**
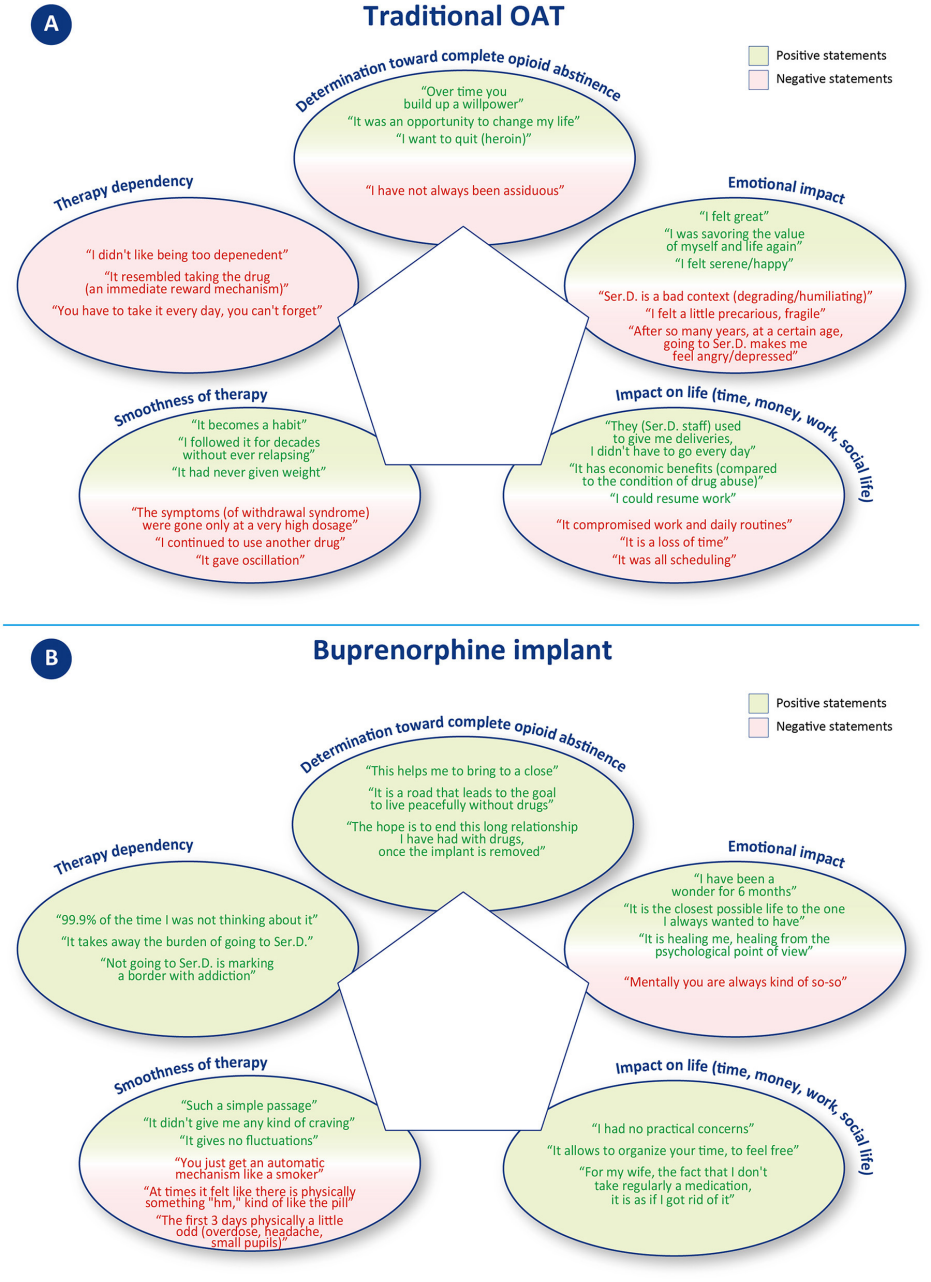
Statement examples included in the narrative analysis. Statements in **(A)** concern traditional OAT; statements in **(B)** concern buprenorphine implant.

In the narrative analysis, the quotations were assigned to one or more of the following categories: 1. determination toward complete opioid abstinence, 2. emotional impact, 3. impact on life (time, money, work, and social life), 4. smoothness of therapy, and 5. therapy dependency. Positive or negative valence was extrapolated for each quotation, based on the expressed narrative. Figures 1A, 2A depict the outcome of the analysis. Each category constitutes a vertex of the pentagon, on which the number of positive (green) or negative (red) iterations per category are plotted and exemplified.

#### 3.2.1. Determination toward complete opioid abstinence

This category includes quotes that expressed motivation or not to remain adherent to drug treatment with oral OAT. Three patients gave a total of eight positive statements (vs. 1 negative). Positive quotes mainly revolved around the desire to quit drugs, to change one's life, and to commit to achieving abstinence; on the contrary, the unique negative comment was about not fully adhering to the oral OAT.

#### 3.2.2. Emotional impact

This category includes quotes that referred to identifiable emotions (e.g., anger, happiness, depression, etc.). Patients gave a total of 9 positive and 19 negative statements. Positive quotes reported emotions such as serenity, happiness, and feeling good. Negative statements, on the other hand, were about a sense of frailty, anger, and depression, relative to still having to attend Ser.D. after many years, and to how this environment is perceived as degrading/humiliating.

#### 3.2.3. Impact on life (time, money, work, and social life)

This category includes quotes that referred to time, money, work, and social life. Patients gave a total of 4 positive and 14 negative statements. Negative statements mainly concerned the organizational difficulties related to having to visit Ser.D. regularly and how this activity took time, forced constant organization, and compromised work and personal routines. On the other hand, positive statements refer to the regained ability to go to work, resulting in a financial income, and to how, in one case, oral OAT was received by delivery, so that the patient did not have to go to the Ser.D. every day.

#### 3.2.4. Smoothness of therapy

This category includes statements that denoted possible intervening problems (including “craving”) or smoothness of events. Patients gave a total of 16 positive and 13 negative statements. Positive statements indicate how oral OAT can become a habit that many of the patients have been able to follow without difficulty for years. Negative comments, on the other hand, concerned cases of patients experiencing a relapse, severe withdrawal syndromes, or other treatment-related disadvantages (desire to smoke and mood swings).

#### 3.2.5. Therapy dependency

This category includes statements that denoted a sense of freedom or dependence toward therapy. No positive statement was given, while a total of 10 negative statements could be identified. These comments referred to the constant need to depend either on someone (sanitary staff providing OAT) or on the medication itself (which gives the same reward mechanism as the drug).

Overall, the narrative analysis of the interview revealed patients' strong intentionality to achieve abstinence from opioids during traditional OAT (8 positive statements vs. 1 negative). A high number of positive statements also emerged in the areas of smoothness of oral OAT therapy, although several negative statements were recorded mainly related to therapy “fluctuations” (16 positive vs. 13 negative). A more negative balance emerges in regard to emotional impact, with 19 negative vs. 9 positive statements. While positive statements referred to a feeling of wellbeing, negative quotes mostly referred to the burden of attending the Ser.D. This issue also emerged in the areas of “impact of life,” with a clear majority of negative statements concerning the loss of time and difficulties in planning, and “therapy dependency,” in which no positive statements were recorded. A summary of patient narratives is available in Appendix II.

### 3.3. Experience with buprenorphine implant—semi-quantitative analysis

The next part of the interview allowed the participant to talk about the proposal and experience with the buprenorphine implant. Among the reasons for accepting buprenorphine implants (Supplemental Table 4), the most common involved being “stable” (in terms of dosage) for a long time. Some patients specifically mentioned their search for a sense of closure and definitive abstinence from opioids, while some showed enthusiasm and also insecurities (i.e., fear to experience craving). Some patients were also driven by practical needs (more time for work and personal life), and one patient reported that he did it out of trust in his treating physician who proposed him the implant.

In the narrative analysis, the quotations were assigned to the same five categories as for traditional OAT: 1. determination toward complete opioid abstinence, 2. emotional impact, 3. impact on life (time, money, work, and social life), 4. smoothness of therapy, and 5. therapy dependency. Positive or negative valence was extrapolated for each quotation, based on the expressed narrative. Figures 1B, 2B depict the outcome of the analysis. Each category constitutes a vertex of the pentagon, on which the number of positive (green) or negative (red) iterations per category are plotted and exemplified.

#### 3.3.1. Determination toward complete opioid abstinence

This category includes quotes that expressed motivation to remain adherent to the treatment to reach abstinence from opioids. Only positive statements were recorded, for a total of 14 positive iterations. Patients primarily cited a desire to progress in their treatment course and to reach a conclusion to their OUD recovery journey.

#### 3.3.2. Emotional impact

This category includes quotes that referred to identifiable emotions (e.g., anger, happiness, depression, etc.). This category received the most iterations of all those examined with a total of 44 statements, 43 of which were positive and 1 was negative. Positive statements described wellbeing, happiness, serenity, lucidity, and renewed ability to enjoy life. The only negative comment concerned occasionally feeling “so-so” from a psychological point of view.

#### 3.3.3. Impact on life (time, money, work, and social life)

This category includes quotes that referred to time, money, work, and social life. Patients only gave positive statements, for a total of 18 positive iterations. The statements mostly referred to the possibility of freely organizing one's own time (vacations, work) and having fewer worries. Some patients also reported receiving positive comments from family members describing a perception of improvement in the patient's status.

#### 3.3.4. Smoothness of therapy

This category includes statements that denoted possible intervening problems (including “craving”) or smoothness of events. Patients gave a total of 21 positive and 5 negative statements. Positive statements referred to the absence of particular physical or psychological variations, a consistent benefit over time. Most patients reported an easy adaptation process, whereas only one describes some side effects in the first 3 days after the implant surgery procedure. In terms of cravings, only one patient described feeling a few physical signs of withdrawal symptoms, whereas at least two patients explicitly referred to not having experienced any cravings.

#### 3.3.5. Therapy dependency

This category includes statements that denoted a sense of freedom or dependence toward therapy. Patients gave a total of 16 positive and 1 negative statements. Positive statements concerned achieving independence from Ser.D. and the consequent possibility of “forgetting” to undergoing treatment. Only one patient mentioned that, either by oral or subcutaneous administration, he remained dependent on a drug/medication.

Overall, patients described mostly positive outcomes from buprenorphine implants. The final balance is positive for all five categories considered, with a peak in regard to emotional impact, which collected the most positive iterations. Only seven negative statements were recorded across categories, of which five were about the smoothness of therapy. A summary of patient narratives is available in Appendix II.

### 3.4. Healthcare context—qualitative analysis

In exploring the outcomes of buprenorphine implant therapy, patients also investigated their relationship with the healthcare professionals who surrounded them. Two patients mentioned the importance of a good clinician–patient relationship and one specified that the implant proposal should come from a trusted physician, who knows the patient's history well. Another patient reported that he was impressed by the care he had received during the surgical procedure and in the follow-up monitoring and the quality of the facility.

In the description of an ideal patient suitable for buprenorphine implant proposal, patients reported that the individual should be pharmacologically and psychologically stable and firm in the intent to abstain from drug abuse, in part because buprenorphine implant will remove the control over the dosage and the “ritual of intake” generally associated with oral OAT. The importance of psychological support during the 6-month treatment with buprenorphine implant is also emphasized, not only on behalf of healthcare professionals but also from stable social relationships (e.g., family and friends) outside of the sanitary context. One patient proposed that the implant should be described as a tool to get closer to the goal of complete opioid abstinence and that if, at the end of the 6 months, the patient wants to go back, this should appear as a plausible solution. A summary of patient narratives is available in Appendix II.

## 4. Discussion

Although sublingual OAT is the cornerstone for treating opioid use disorder, its management can be challenging due to its impact on daily routine, social stigma, and the risk of misuse and diversion (4, 9, 10). Innovative long-acting formulations, such as buprenorphine implants, can ensure the optimization of patient management by improving adherence to therapy and quality of life (4, 10). In this study, we present the experience of some of the first patients in Europe who received buprenorphine implants and were interviewed using a semi-guided track. This study compares the experiences of five OUD patients before and after the introduction of a new treatment approach via buprenorphine implant. Using a combination of qualitative and semi-quantitative approaches, we identified recurring themes and assessed the strengths and disadvantages of traditional OAT and buprenorphine implant therapy. A summary of patient narratives is available in Appendix II.

Patients expressed positive determination to achieve complete opioid abstinence in both treatment strategies, with 8 positive statements (vs. 1 negative) for traditional OAT and 14 positive statements for buprenorphine implant. Most of the patients described buprenorphine implant as a further step toward resolving their addiction journey: “This helps me to bring [sic]to a close,” “The hope is to end this long relationship I have had with drugs, once the implant is removed.” The patients' enthusiasm toward the buprenorphine implant proposal and their strong determination to achieve complete opioid abstinence are significant factors that favor good adherence to therapy and ultimately lead to a positive treatment outcome. According to their own words, buprenorphine implant allows forgetting about the ongoing therapy or even scheduled removal, contributing to the re-establishment of a new “normality.” According to one patient, this is “the closest life to that of non-drug addicts,” and another describes it as “psychologically curative.”

Patients' experience with traditional OAT was marked by a significant improvement in their previous condition of substance abuse (“I was savoring[sic] again the value of myself and life”; “I felt great”). However, there were also practical and emotional challenges hindering the achievement of a new “normality.” Emotional impact, for instance, emerged as a key issue, with patients reporting 9 positive and 19 negative statements, the latter describing a sense of frailty, anger, and depression, particularly in relation to still having to attend Ser.D., especially after many years. Several patients described Ser.D. as a “degrading” context, which further contributed to fueling negative emotions. On the other hand, patients who received buprenorphine implants reported improved social relationships, a closer bond with family members, and reduced stigma. Emotional impact was the most frequently discussed category over all interviews, with a total of 44 statements, of which only one was negative. Positive emotions included a sense of wellbeing, happiness, serenity, lucidity, and a renewed ability to enjoy life.

Along with the emotional effects, traditional OAT can present practical challenges that impact patients' daily lives, including work, finances, and time management. During the interviews, patients shared 14 negative statements (vs. 4 positive) that mainly related to the organizational difficulties of regular Ser.D. visits, which required significant time, forced constant organization, and disrupted work and personal routines. In contrast, buprenorphine implant offers greater freedom from these constraints, resulting in significant time and management independence (including the ability to organize off-site activities). Patients expressed only positive statements in this concern, with a total of 18 positive iterations.

Overall, patients expressed satisfaction with the smoothness of traditional OAT, with 16 positive and 13 negative statements. However, the cyclic nature of oral OAT can lead to mood and efficacy fluctuations, which may cause relapses and cravings. In contrast, patients on the subcutaneous implant, a long-acting formulation that releases buprenorphine gradually and constantly, reported consistent benefits and did not encounter any specific difficulties in adapting to the new therapy. In this context, patients provided 21 positive and 5 negative statements. It is worth noting that patients typically receive buprenorphine implants after undergoing pharmacological and psychological stabilization, suggesting that these two therapies may be appropriate at different stages of the treatment process.

In terms of therapy dependency, oral OAT patients expressed no positive statements and 10 negative statements, indicating a constant need to depend either on the medication or on healthcare providers. On the other hand, buprenorphine implant patients had a more positive outlook, with 16 positive statements and only 1 negative statement. Patients reported achieving independence from healthcare providers and the ability to “forget” about undergoing treatment. However, one patient did mention remaining dependent on a drug/medication, regardless of the mode of administration. It is important to note that the sense of therapy dependency can be influenced by individual patients' experiences and perceptions.

A last consideration concerns the ideal patient profile. Given the heterogeneity of the sample of patients in terms of social background and history of substance abuse (including the type of drugs abused, duration, and context), a buprenorphine implant appears to be a suitable treatment option for a wide range of individuals. Patients themselves have emphasized the importance of pharmacological, psychological, and social stability, as well as the doctor–patient relationship, as crucial factors in determining the suitability of the treatment.

In conclusion, the buprenorphine implant emerges as a viable therapeutic option for improving the quality of life of eligible patients who are favorable to undergoing such therapy. The buprenorphine implant is specifically indicated for the treatment of OUD in clinically stable, opioid-tolerant adult patients who require a maximum of 8 mg/day of sublingual buprenorphine as OAT. However, it is worth noting that patient acceptance of this therapy has a critical role in determining the success of the treatment (25), as it introduces changes to the patient's treatment routine and involves a minor surgical procedure. Therefore, it is of utmost importance to provide patients with sufficient information about the treatment process with the buprenorphine implant. Moreover, comparative studies have shown that the use of buprenorphine implants does not result in a lower likelihood of maintaining therapy response compared to continued sublingual buprenorphine intake. Over 6 months, 72 out of 84 patients (85.7%) receiving buprenorphine implants and 64 out of 89 patients (71.9%) receiving sublingual buprenorphine maintained opioid abstinence (hazard ratio, 13.8; 95% CI, 0.018–0.258; *P* = 0.03) (26). Furthermore, long-lasting buprenorphine administration was found to be well-tolerated, with a safety profile consistent with that of sublingual buprenorphine (27). Finally, a systematic benefit–risk assessment, utilizing a semi-quantitative method that considers the risk of diversion and misuse, treatment compliance, quality of life, adverse effects, and surgery-associated risks, has found the profile of the buprenorphine implant to be favorable when compared to sublingual buprenorphine (28).

## 5. Psychological considerations

The cornerstone of the experience of users who have undergone buprenorphine subcutaneous implant seems to be resuming control over one's own life. Tied to the assumption of buprenorphine or methadone, in their imagination, they were too often associated with the vision of the “lifelong addict”: now, they can see themselves as men and women freed from daily procedural constraints. This perspective shift could be constructed, or rather co-constructed, thanks to a relationship of trust based on authenticity and clarity. Working on breaking down stigma represented an essential issue for good therapeutic compliance and the functional acceptance of the implant as part of the self and not as a “foreign body.” The body emerged as another highly transformative psychological element. If in classical assumptions—whether methadone or buprenorphine, as well as in (and even more so) the use of narcotic substances—the user used to reserve passive attention to the body, as if it were a container, now a renewed bodily experience seems to emerge. The implant, accepted as a body part and secreting what the user needs, almost without realizing it, restores a sort of “therapeutic pride” as if to say “I am indeed the actor of my own state of health.” This last symbolically important concept pushes us to further explore, through interviews and psychotherapies, the extent and impact at multiple levels that the implant has on users and their overall quality of life, reaffirming that it constitutes an opportunity for healthcare professionals who wish to use it as a relational tool in the care of our patients.

## Data availability statement

The raw data supporting the conclusions of this article will be made available by the authors, without undue reservation.

## Ethics statement

Ethical review and approval was not required for the study on human participants in accordance with the local legislation and institutional requirements. The patients/participants provided their written informed consent to participate in this study.

## Author contributions

All authors listed have made a substantial, direct, and intellectual contribution to the work and approved it for publication.
