# Supplementary material for: The experience of buprenorphine implant in patients with opioid use disorder: a series of narrative interviews
Source: Front Psychiatry. 2023 Aug 31;14:1205285. doi: 10.3389/fpsyt.2023.1205285 (PMC10501400; doi:10.3389/fpsyt.2023.1205285)
Supplement: Supplementary file 1 [file Table_1.DOCX]

**Supplemental table 1. Summary of participants’ childhood experience**

| **CHILDHOOD** | **Positive feelings** | **Negative feelings** |
| --- | --- | --- |
| References to school (including sociality) | N of patients = 4 | N of patients = 1 |
|  | Examples: “I had good grades”, “an excellence that would fill the family illiteracy” | Examples: “three horrible years of middle school as compared to nice elementary and high schools” |
| References to family | N of patients = 4 | N of patients = 1 |
|  | Examples: “very normal”, “nice”, “culturally ahead of time”, “close-knit, […] with numerous convivial occasions” | Examples: “my father is busy managing the current and former families” |
| References to (general) happiness/unhappiness | N of patients = 3 | N of patients = 1 |
|  | Examples: “my childhood went quite well”, “as a child I was happy”, “I had self-confidence” | Examples: “I was suffering” |
| References to normality/discrimination | N of patients = 3 | N of patients = 1 |
|  | Examples: “normality”, “up to age 19 everything went quietly”, “friends saw me for who I was and not what I looked like” | Examples: “I felt treated differently… I was often singled out” |

Note: each patient (total N = 5) explored these themes freely and mentioned one or more words related to these categories; the interview was created to elicit responses on each of these themes. Patients were assigned to each category only when an explicit mention could be recorded.
