# Supplementary material for: The experience of buprenorphine implant in patients with opioid use disorder: a series of narrative interviews
Source: Front Psychiatry. 2023 Aug 31;14:1205285. doi: 10.3389/fpsyt.2023.1205285 (PMC10501400; doi:10.3389/fpsyt.2023.1205285)
Supplement: Supplementary file 2 [file Table_2.DOCX]

**Supplemental table 2. Summary of patients' context of drug of abuse initiation**

| **CONTEXT OF DRUG USE INITIATION** | **N of patients** |
| --- | --- |
| Mental distress/suffering | N of patients = 3 |
|  | Examples: “I had a psychological breakdown”, “there was something I was suffering for, without clear identification”, “it relieved pain, the hidden pain, the pain of the soul” |
| Naivety/lack of knowledge | N of patients = 2 |
|  | Examples: “I started without giving too much importance”,  “I was completely unprepared”, “I had never heard of it” |
| For fun/discovery | N of patients = 2 |
|  | Examples: “I tried them all, to see the kind of effect they had”, “I did it for fun, to try” |
| Driven by social environment | N of patients = 2 |
|  | Examples: “I met the underground world”, “my close acquaintances were using drugs” |
| Intentional search | N of patients = 1 |
|  | Examples: “I did it less for fun, more by choice”, “it felt like something separate from the rest of my life” |
| To feel different (from others) | N of patients = 1 |
|  | Examples: “I did it to feel rebellious, nonconformist” |

Note: each patient (total N = 5) explored these themes freely and mentioned one or more words related to these categories; the interview was created to elicit responses on each of these themes. Patients were assigned to each category only when an explicit mention could be recorded.
