# Supplementary material for: The experience of buprenorphine implant in patients with opioid use disorder: a series of narrative interviews
Source: Front Psychiatry. 2023 Aug 31;14:1205285. doi: 10.3389/fpsyt.2023.1205285 (PMC10501400; doi:10.3389/fpsyt.2023.1205285)
Supplement: Supplementary file 3 [file Table_3.DOCX]

**Supplemental table 3. Summary of patients’ major consequences of drug abuse**

| **CONSEQUENCES OF DRUG ABUSE** | **N of patients** |
| --- | --- |
| Despair/suffering/breakdown | N of patients = 4 |
|  | Examples: “I felt bad”, “you find yourself absorbed […] going down”, “an intolerable situation" |
| Sense of dependence | N of patients = 3 |
|  | Examples: “after a few months it is no longer a choice [...] it had become a necessity, otherwise I was sick”, “you have to look for it *…* it is a vicious circle*”*, *“*you feel like a slave*”* |
| Job loss or other professional/school problems | N of patients = 2 |
|  | Examples: “I lost my job”, “even in a moment of beautiful achievement (graduation), I was shielded” |
| Relational issues | N of patients = 2 |
|  | Examples: “at first one feels euphoric then one shuts down [...] you don't socialize anymore, and if you socialize you do it with the wrong people”; “people get fed up and move away […] everything moves away” |
| Economic problems | N of patients = 2 |
|  | Examples: “you don't have any more money” |

Note: each patient (total N = 5) explored these themes freely and mentioned one or more words related to these categories; the interview was created to elicit responses on each of these themes. Patients were assigned to each category only when an explicit mention could be recorded.
