# Supplementary material for: The experience of buprenorphine implant in patients with opioid use disorder: a series of narrative interviews
Source: Front Psychiatry. 2023 Aug 31;14:1205285. doi: 10.3389/fpsyt.2023.1205285 (PMC10501400; doi:10.3389/fpsyt.2023.1205285)
Supplement: Supplementary file 4 [file Table_4.DOCX]

**Supplemental table 4. Summary of patients’ reasons for accepting buprenorphine implant**

| **IMPLANT PROPOSAL/ACCEPTANCE** | **N of patients** |
| --- | --- |
| I was in a stable moment (also in terms of dosage) | N patients = 3 |
|  | Examples: “I was at a good level”; “I was in the same dose since a long time” |
| I was driven by a sense of ultimate conclusion/independence/perspective to quit | N patients = 3 |
|  | Examples: “I wanted to take it off completely”, “It is a path to the goal (living peacefully without substances/drugs)” |
| I was driven by practical needs (time, work, personal life) | N patients = 2 |
|  | Examples: “I was driven by the improvement of quality of life, gain of time”, “No longer being forced to come to Ser.D.” |
| I felt enthusiasm/curiosity/interest in trying this new formulation, curiosity, interest in undertaking treatment | N patients = 2 |
|  | Examples: “I was happy of this opportunity”, “It tantalized me so much” |
| I had fears/insecurities | N patients = 2 |
|  | Examples: “When I put the implant in, I was terrified (I go through withdrawal, I feel sick)”, “I was afraid it meant taking a shortcut/easy way” |
| I trusted the person who proposed it to me | N patients = 1 |
|  | Examples: “It was proposed to me by a psychotherapist in whom I have immense confidence, a few words the right ones” |

Note: each patient explored these themes freely and mentioned one or more of these categories; the interview was not guided in order to elicit responses on each of these themes. Patients were assigned to each category only when that was explicitly mentioned.
