# Supplementary material for: The experience of buprenorphine implant in patients with opioid use disorder: a series of narrative interviews
Source: Front Psychiatry. 2023 Aug 31;14:1205285. doi: 10.3389/fpsyt.2023.1205285 (PMC10501400; doi:10.3389/fpsyt.2023.1205285)
Supplement: Supplementary file 5 [file Data_Sheet_1.docx]

**Appendix I. Full text of the semi-structured interview**

**Instructions: Below you will find a few questions for the purpose of giving voice to your story and emotions about your experience with drugs, with traditional daily OAT, and your current experience with the subcutaneous buprenorphine implant. Your narrative will be entirely anonymous and is valuable as it will help create a more complete and better picture of the healing experience you and other users have undergone.**

**During your narrative, please never mention the name of the active ingredient or the trade name of the drug you were given, but always refer to this therapy by mentioning the words subcutaneous implant.**

***Brief life history***

- Go back in time and try to tell your story before "drugs" entered your life. What kind of child were you, how did you experience school, how were you viewed by your family, friends, etc.?
- What impact did the encounter with "drugs" have into your life? Can you recount how it happened, with whom and in what way did you use it (frequency, mode of intake, etc.)?
- If you look back at your past, are the motivations that pushed you and made you stay in the opioids world clearer today?
- What were the consequences of opioids use in your personal, family, social, and work relationships?

***Encounter with Ser.D. and traditional therapies***

- What led you to approach the Addiction Service? What expectations did you have (reason, triggering event, need, usefulness)?
- Regarding traditional daily OAT, what can you tell us? How long did you follow it for?
- What were the benefits and limitations, emotions, effects, and repercussions in daily, family, and work life due to the use of traditional therapy?
- Was it included in an individual, couple, family, or group psychotherapeutic course? Or did you essentially follow only a medical directive?

***Ongoing experience with the subcutaneous buprenorphine implant***

- In what context and who first introduced the subcutaneous buprenorphine implant to you, and how was it described?
- What did you think about this proposal and what made you agree to undergo this treatment?
- How was your perception of the first few days after the subcutaneous buprenorphine implant? Were you able to adapt easily to the change?
- When you think about your social life and your emotions, in your daily life, what differences do you find between traditional oral OAT and the subcutaneous implant? In particular, what is your opinion, compared to the previous therapy, regarding ease of adherence to therapy, practicality, daily autonomy, and any other effects you can think of?
- Has your perception of yourself or how you are viewed by others changed? Do you feel less ‘addicted’ than when you were using traditional OAT?
- Have you happened to talk about this subcutaneous implant to people with a history of addiction similar to yours? Have you discussed it with your family members, if so, what did they tell you, and if not, why not?
- How would you explain how the subcutaneous implant works and on what areas of your life do you feel the greatest benefit?

***Conclusive remarks***

- What have you learned from your clinical journey that might be helpful to other patients and to health care providers themselves? Why would a health care provider suggest the use of this subcutaneous implant to a patient?
- What kind of people could benefit from this subcutaneous implant?
- What human and professional features should health professionals possess when suggesting the subcutaneous implant to other interested people?
- Do you think that this implant could have long-term effects in your process of getting rid of substance use?
- What are your expectations about its future today? In particular, how do you think the subcutaneous implant will help you to realize them?

  Thank you for your time and for your valuable feedback.
