# Supplementary material for: The experience of buprenorphine implant in patients with opioid use disorder: a series of narrative interviews
Source: Front Psychiatry. 2023 Aug 31;14:1205285. doi: 10.3389/fpsyt.2023.1205285 (PMC10501400; doi:10.3389/fpsyt.2023.1205285)
Supplement: Supplementary file 6 [file Data_Sheet_2.docx]

**Appendix II. Summary of patients’ narratives**

**Patient 1**

Patient 1, of African descent, reports growing up in a “*very normal*” and “*important*” foster family who enrolled him in “*private multi-ethnic schools*”. He reports that he sometimes perceived discrimination towards him but, talking about his friends, he mentioned that they used to “*see me for who I was and not how I looked*”. He started using illegal drugs at the age of 30, due to a “*psychological breakdown*” after separating from his wife. He used drugs for about 18 years, although “*not always regularly*”; he also reports occasional use of cannabis.

The patient reports deciding to attend the Ser.D. partly because he no longer had the finances to buy heroin (“*to make a quick fix*”) and partly because of the addiction’s negative effects on his relational and occupational life (“*I lost my job*”). The patient underwent OAT for about 15 years, including a period in a rehab centre. He continued to perceive himself as “a patient” because of the regular visits to the Ser.D. to receive OAT, and this had a negative impact on his social and work relationships.

The treating clinician at Ser.D. evaluated the patient’s path and his commitment and proposed him the subcutaneous buprenorphine implant (“*I earned a recognition after ten years*”). The patient accepted mainly because he was tired of regularly going to the Ser.D. to receive OAT (“*It was becoming depressing*”). The implant became an opportunity to save time for family, work, and personal matters. The first few days after the implant, he felt “*very well*”, without discomfort or craving. The patient reports that, now that he has the subcutaneous implant, his “*brain is recovering*”, and he is regaining lucidity. Other people’s perception of him has also changed, particularly among family members.

The patient describes the subcutaneous implant as “*a bolt from the blue*”: he found it to be “*curative*” also from a psychological point of view and was amazed by its immediate effect on his life, after more than 17 years of addiction. Nowadays, he feels free from the burden of the frequent follow-up visits (except for those necessary to monitor the implant), he reports feeling physically and mentally well, and that he has regained his longed-for serenity.

**Patient 2**

Patient 2 reports that he was an introverted and sensitive child who grew up in a family “*culturally a step ahead of times, who understood the speed of a society that was evolving*”. He was very good at school, and the family perceived him as “*an excellence that would have filled their illiteracy*”. The patient started using cannabis during adolescence, first occasionally, then daily, and then he switched to heroin, “*which relieved the pain, the hidden pain, the pain of the soul. […] A great love... too much so*”.

He started to attend the Ser.D. expecting to find “*the formula that would extinguish the heroin ardour*”, thus to “*save*” himself. The patient had followed OAT for about ten months, accompanied by group and individual psychotherapy. After experiencing the reduced quality of life with oral OAT, he agreed to undergo the subcutaneous implant proposed by his treating clinician, since the oral OAT “*took up time*” and especially because “*at my age, [being dependent on the Ser.D.] made me feel angry*”, in addition to the perceived strong stigma. Attending the Ser.D. represented a mere “*boundary on the borderline of addiction*”. Independence represented the main difference from oral OAT and the greatest benefit of the implant (“*you feel freer*”) and the “*rage*” of having to attend the Ser.D. disappeared.

The patient emphasises the consistency of the implant’s benefits over time, especially in the emotional sphere: “*No emotional rebound, no spike like when taking the tablet, no collapse*”. He also describes it as “*an excellent deterrent since the buprenorphine-illegal opioid combination is dangerous*”.

**Patient 3**

Patient 3 reports a “*very normal childhood*”, in which he did not suffer much of the critical aspects of his parents’ divorce. He remembers a very hard-working father, a good relationship with his father’s previous marriage family, and three years of “*horrible*” middle school, as compared to regular primary school. He reports having tried heroin after some sporadic use of cannabis, first for fun, then occasionally with his best friend and finally regularly, even on his own, between the end of the first and the beginning of the second year of university.

When he started to increase his heroin use, he began to have financial difficulties, as well as “*damage that was accumulating in various aspects of my life*”, which prompted him to take “*a next step*”, which was to go to the Ser.D. after an initial individual psychiatric therapeutic path. Switching to OAT represented for him taking action and being able to prove to his family his commitment. However, the patient initially used illegal substances concomitantly with OAT, before finally quitting. Going to the Ser.D., once a week first, twice a week later, entailed “*fatigue*” for him: he perceived this therapy just as a “*replacement*” of the subject of his addiction.

The treating clinician at the Ser.D. proposed the subcutaneous implant to the patient, which he promptly accepted mainly because of the possibility of reducing the dosage. However, he was concerned about losing the habit of the daily intake, whereby the immediate reward associated to this “*ritual*” moment. Although he was nervous about the surgery, the first impact of the implant was positive, as he had the perspective of potentially being able to completely quit drugs. The first few days after the implant insertion, he felt “*very well and serene*”. The implant allowed him to “*distract himself*” and feel secure to the extent that he forgot that he was meant to remove it at the sixth month.

For this patient, the implant represents a fundamental element of his recovery pathway. According to him, the implant removes the control of the dosage and the “*ritual*” of the daily intake, particularly in the perspective of not relapsing to drug abuse upon implant removal.

**Patient 4**

Patient 4 describes the family of origin as “*very normal*” and close-knit and reports a comfortable childhood and numerous familiar convivial occasions. The patient reports having tried heroin in his early 30s during a business trip to Afghanistan, after previous occasional cannabis use. After ten years of heroin abuse, the patient reports that he “*quitted*” for three years and relapsed during a further trip to an Asian country.

On his return to Italy, the patient started attending the Ser.D., where he started OAT with methadone and then switched to sublingual buprenorphine. The patient has remained on buprenorphine 8 mg for 23 years and reports no particular issues with this therapy. However, attending the Ser.D. every 15 days was inconvenient, as he had to find a suitable time during working hours to visit the facility. In particular, he points out that attending Ser.D. had become “*a habit*”, a substitute for his addiction.

The patient learned about the subcutaneous implant from his treating clinician and accepted to undergo this treatment. He did not experience the withdrawal symptoms upon buprenorphine tablet discontinuation in preparation for surgery. He reports feeling “*wonderfully*” at the moment of the interview. In addition to the advantage reported by other patients, he reports not feeling the urge to smoke that used to accompany his daily oral OAT intake.

The patient reports that his experience with the subcutaneous implant is very positive, mentioning that he forgot that he had to remove it. He also reports that his wife is happy with this result and that, although “*the substance is still under the skin*”, he feels free, also from social stigma. He is finally leading a normal life and hopes to “*finally end this long relationship with drugs*” after six additional months with a second implant.

**Patient 5**

Patient 5 reports having had a happy childhood, and his life *"went smoothly"* until the age of 19. After moving to a new city, he discovered *"the underground world"* where many drugs were circulating, and he wanted to *"try them all, to experience which kind of effect they were giving".* Opium became his drug of choice, and he describes a feeling of invincibility that led him to pursue its use. However, *"after a few months, the search for opium was no longer a choice, but it had become a necessity, otherwise I was feeling sick."*

The patient committed to the Ser.D. and started a buprenorphine sublingual therapy. He reached a high dosage (28 mg) before withdrawal symptoms could disappear; when this happened, he *"cried all day out of happiness".* The therapy continued for several years (*"I strengthened myself", "My life was going well"*), albeit with some occasional issues (*"After 7 years, I started craving again"*). The patient reports a sense of "fluctuation" with the oral therapy *("I was hyperactive in the morning, then I would shut off in the afternoon"*), as well as some discomfort with the Ser.D. *("It was just a dispenser", "An unpleasant reality," "It didn't allow me to make plans"*); furthermore, the patient states that *"daily intake make you feel as if you are sick".* After a long period at 2 mg of sublingual buprenorphine – during which *"everything was going well" –* the patient felt a desire to further reduce the dosage but did not know how to.

With these premises, he accepted his doctor’s proposal to undergo the buprenorphine implant therapy; he considered this as *"a chance leading to the goal of living peacefully without drugs or substances"* ("*A solution that makes you forget*"). The patient reports having been *"closely monitored",* and – apart from the first three days after surgery being "*physically a bit strange*" – he felt good (or "*very good*"), "*enjoying”* himself and "*free*". The patient frequently refers to the topic of freedom, using expressions such as *"Feeling free to plan",* "*Independent of drug intake*", "*Not being bound by the obligation to visit the Ser.D.*", "*A taste of what life would be like without the implant*". He also mentions the absence of fluctuations associated with oral therapy ("*I no longer have swings*"). Finally, this patient repeatedly states how life with the buprenorphine implant “*is as close as possible to that of non-addicted individuals, the life I always wanted to have, to face life almost on equal terms than others*”.
